# Correlation analysis of monocyte chemoattractant protein-1 and clinical characteristics and cognitive impairment in type 2 diabetes mellitus comorbid major depressive disorder

**DOI:** 10.3389/fnagi.2023.1081393

**Published:** 2023-05-04

**Authors:** Fang Cao, Mei Yang, Yuqi Cheng, Xiuyue Zhang, Li Shi, Na Li

**Affiliations:** ^1^The First Affiliated Hospital of Kunming Medical University, Kunming, Yunnan, China; ^2^The Third People’s Hospital of Yunnan Province, Kunming, Yunnan, China; ^3^The First People’s Hospital of Yuxi, Yuxi, Yunnan, China; ^4^Department of Endocrine, The First Affiliated Hospital of Kunming Medical University, Yunnan, China

**Keywords:** type 2 diabetes mellitus comorbid major depressive disorder, monocyte chemoattractant protein-1, clinical characteristics, cognitive impairment, pathophysiology

## Abstract

**Introduction:**

Type 2 diabetes mellitus (T2DM) and major depressive disorder (MDD) are both chronic diseases, and they are often co-morbid. Usually, T2DM and MDD are associated with cognitive impairment, and the comorbidity status of both may increase the risk of cognitive impairment, but the underlying pathogenesis is not clear. Studies have shown that inflammation, especially monocyte chemoattractant protein-1 (MCP-1), could be associated with the pathogenesis of type 2 diabetes mellitus comorbid major depressive disorder.

**Aims:**

To investigate the correlations of MCP-1 with clinical characteristics and cognitive impairment in type 2 diabetes mellitus patients combined with major depressive disorder.

**Methods:**

A total of 84 participants were recruited in this study, including 24 healthy controls (HC), 21 T2DM patients, 23 MDD patients, and 16 T2DM combined with MDD (TD) patients, to measure the serum MCP-1 levels using Enzyme-linked Immunosorbent Assay (ELISA). And the cognitive function, depression, and anxiety degree were assessed using Repeatable Battery for the Assessment of Neuropsychological Status (RBANS), 17-item Hamilton Depression Scale (HAMD-17), and Hamilton Anxiety Scale (HAMA), respectively.

**Results:**

(1) Serum MCP-1 expression levels in the TD group were higher than HC, T2DM, and MDD groups, respectively (*p* < 0.05). And compared with HC and MDD groups, serum MCP-1 levels in the T2DM group were higher (*p* < 0.05) statistically. Receiver Operating Characteristic (ROC) curve showed that MCP-1 could diagnose T2DM at cut-off values of 503.8 pg./mL (sensitivity 80.95%, specificity 79.17%, AUC = 0.7956) and of 718.1 pg./mL for TD (sensitivity 81.25%, specificity 91.67%, AUC = 0.9271). (2) Group differences in cognitive function were significant. Compared with the HC group, total RBANS scores, attention scores, and language scores in the TD group were lower, respectively (*p* < 0.05), and total RBANS scores, attention scores, and visuospatial/constructional scores in the MDD group were lower, respectively (*p* < 0.05). Compared with the T2DM group, immediate memory scores in HC, MDD, and TD groups were lower, respectively, and total RBANS scores in TD were lower (*p* < 0.05). (3) Correlation analysis showed that hip circumference was negatively correlated with MCP-1 levels in the T2DM group (*R* = −0.483, *p* = 0.027), but the correlation disappeared after adjusting age and gender (*r* = −0.372; *p* = 0.117), and there were no significant correlations between MCP-1 and other variables.

**Conclusion:**

MCP-1 may be involved in the pathophysiology of type 2 diabetes mellitus patients combined with major depressive disorder. And MCP-1 may be significant for the early evaluation and diagnosis of TD in the future.

## Introduction

Chemokines are a family of small, signaling proteins secreted by the cells of the immune system, with a small molecular weight of 8–14 kDa. The chemokine family is divided into four families with two major subgroups (CXC and CC) and two smaller subgroups (CX3C and C). And Monocyte chemotactic protein-1 (MCP-1) belongs to the CC family. MCP-1 triggers the chemotaxis and transendothelial migration of monocyte to the inflammatory places by interacting with the membrane CC chemokine receptor 2 in monocytes (Koth et al., 2004; Singh et al., 2021).

Type 2 diabetes mellitus is a condition in which the body’s cells do not respond well to the hormone insulin on the background of insulin resistance, and accounts for 90–95% of all diabetes mellitus in adults (American Diabetes Association, 2020). Mechanism of inflammation has been shown important potential in assessing the risk of diabetes mellitus and its complications (Muzurović et al., 2021). Proinflammatory factors produced from a chronic inflammatory state, such as MCP-1, may promote the development of T2DM by increasing insulin resistance in peripheral tissues like the liver, muscle, and adipose tissue (Hotamisligil, 2017), and anti-inflammatory treatment had beneficial effects on glucose metabolism and cardiovascular complications of diabetes (Donath, 2014). In addition, some studies have found that peripheral MCP-1 levels increased in T2DM patients, and MCP-1 expression levels were associated with insulin resistance (Degirmenci et al., 2019; Muzurović et al., 2021). MCP-1 was also involved in the development of T2DM complications, and MCP-1 significantly elevated in patients with T2DM retinopathy, osteoporosis, cardiovascular complications, and diabetic nephropathy (Reddy et al., 2017; Ferland-McCollough et al., 2018; Wang et al., 2019; Schrauben et al., 2021). Furthermore, MCP-1 was associated with the response to the treatment of diabetes, but MCP-1 did not appear to be significantly correlated with HbA1c, fasting glucose, and postprandial glucose (Kher et al., 2020). What’s more, cognitive dysfunction, including mild cognitive impairment and dementia, was also increasingly recognized as an important comorbidity and complication of diabetes (Biessels and Whitmer, 2020). A big data study showed that the prevalence of cognitive impairment and/or dementia was 13.1% in diabetic patients aged 65–74 years, and 24.2% in patients over 75 years (Feil et al., 2011). A longitudinal study following up for 10 years showed that higher levels of inflammatory markers such as serum interleukin-6 (IL-6) and C reactive proteins (CRP) were associated with subsequent cognitive decline in older adults with type 2 diabetes (Sluiman et al., 2022).

MDD is a common comorbidity in patients with T2DM, and approximately 28% of patients with diabetes have varying degrees of MDD (Khaledi et al., 2019). Furthermore, patients with T2DM are 1.5–2.0 times more likely to develop a major depressive disorder than non-diabetic patients (Wang et al., 2019). MDD often increases the risk of diabetes, causes poor control of blood glucose levels, and increases the risk of various complications. Conversely, patients with diabetes are also at an increased risk of comorbid MDD, which may increase the severity of MDD and cognitive decline, and affect the antidepressant efficacy and social function of patients (Demakakos et al., 2017). In addition, MDD and T2DM were independently associated with a greater risk of dementia, and the risk of comorbidity of both was greater than the additive risk of the two diseases. Thus, TD is associated with a greater risk of dementia (Katon et al., 2015). TD was also associated with poor diabetes outcomes and poor quality of life (Rauwerda et al., 2018); patients with TD have a higher mortality risk than those with both diseases alone, with a hazard ratio of 2.33 (Castro-Costa et al., 2019). It is therefore essential for the adequate diagnosis and treatment of TD.

Similarly, type 2 diabetes mellitus patients combined with major depressive disorder had a higher level of serum MCP-1 (Nguyen et al., 2021), and serum MCP-1 levels in Alzheimer’s disease patients were higher than those with mild cognitive dysfunction and healthy controls, and the serum MCP-1 levels were the highest in patients with severe Alzheimer’s disease, suggesting that higher serum MCP-1 levels were associated with more severe cognitive impairment (Lee et al., 2018).

Therefore, this research intends to study the impacts of MCP-1 on clinical characteristics and cognitive function, and the correlations between MCP-1 with clinical characteristics and cognitive function in TD patients. Our study would contribute to providing ideas for the early clinical prediction or diagnosis of cognitive impairment of TD.

## Materials and methods

### Participants

A total of 84 participants were included in the present study, including 24 healthy controls, 21 T2DM patients, 23 MDD patients, and 16 TD patients. All participants were recruited from the psychiatric and endocrinology inpatient and of the First Affiliated Hospital of Kunming Medical University during the period from August 2020 to December 2021(*x̄* ± *s*). This study was approved by the Medical Ethics Committee of the First Affiliated Hospital of Kunming Medical University. Informed consent was obtained from all participants.

All participants are aged 18–70 years, the depressive episode and type 2 diabetes were diagnosed according to the Diagnostic and Statistical Manual of Mental Disorders (5th Edition) (DSM-5), and the HAMD-17 scores are equal or greater than 17 for MDD and TD groups, less than 17 for HC and T2DM groups. MDD patients had no previous diagnosis of any type of diabetes mellitus, impaired fasting glucose, or abnormal glucose tolerance, and no previous depressive disorder was diagnosed. TD patients met the diagnostic criteria of DSM-5 for both depressive episode and type 2 diabetes.

Exclusion criteria: (1) Previous intellectual disability, schizophrenia, obsessive–compulsive disorder, bipolar disorder, or any other mental disorder patients were excluded; (2) Previous history of abuse or dependence on psychoactive substances were excluded; (3) Serious organic brain diseases or somatic diseases, including but not limited to cerebral infarction, cerebral hemorrhage, Parkinson’s disease, hypothyroidism, coronary heart disease, tumor, autoimmune diseases were excluded; (4) Patients who were unable to cooperate with all research procedures were excluded.

### Clinical data collection

The demographic data (e.g., gender, age, disease duration, and prior health) and general information (e.g., height, body weight, body mass index, waist circumference, hip circumference, waist-hip rate, systolic pressure, and diastolic blood pressure) were obtained from electronic medical records, direct communication with patients and their families, or on-site measures.

The laboratory characteristics, including total cholesterol (TC), triglycerides (TG), high-density lipoprotein (HDL), low-density lipoprotein (LDL), fasting blood glucose (FBG), fasting insulin (INS), and glycosylated hemoglobin (HbA1c), were tested in the laboratory of the First Affiliated Hospital of Kunming Medical University by collecting fasting blood samples.

Calculation formula: body mass index (BMI) = body weight (kg)/height^2^ (m^2^), insulin resistance index = fasting blood glucose × fasting insulin/22.5, waist-hip ratio = waist circumference/hip circumference.

The cognitive function, depression, and anxiety degree were assessed using Repeatable Battery for the Assessment of Neuropsychological Status (RBANS), 17-item Hamilton Depression Scale (HAMD-17), and Hamilton Anxiety Scale (HAMA), respectively. RBANS includes 12 subtests as vocabulary learning, story retelling, graphic copying, semantic fluency test, number breadth, encoding test, vocabulary recall, vocabulary recognization, story recall, graphic recall, line angle, and picture naming. And RBANS were evaluated from five aspects of cognitive function, including immediate memory (vocabulary learning, story retelling), delayed memory (vocabulary recall, vocabulary recognization, story recall, graphic recall), attention (number breadth, encoding test), language (semantic fluency test, picture naming), and visuospatial/constructional test (graphic copying, line angle). Each cognitive function index was transformed into the standard scores from the original coarse scores using age-stratified regular model tables (Olaithe et al., 2019).

### ELISA assays

Five-milliliter of the whole blood sample was collected from each participant, and all samples were centrifuged for 15 min at 1,000 × g, then the serum was immediately stored at −80°C until further analysis. Serum MCP-1 expression levels were measured using ELISA kits (Elabscience, China) following the manufacturer’s instructions and standard procedures.

### Statistical analysis

Statistical analysis was performed with the statistical package for the social sciences (SPSS) version 26.0. Data were presented by mean ± standard deviation () when the data were normally distributed, and one-way ANOVA test was used for the test of significance. Otherwise, we used the Median (25th percentile–75th percentile) to present non-normal data, and a non-parametric Kruskal-Wallis test was used for the test of significance. Our data of MCP-1 were non-normal data, so we compared MCP-1 levels of study groups using the non-parametric Kruskal-Wallis test. In addition, we performed Spearman correlation analysis to examine the correlation of variables and MCP-1. To reduce the influence of confounding factors, the partial correlation was used by adjusting age and gender for further correlation analysis if there was a correlation between the two variables. Additionally, the receiver operating characteristic (ROC) curve was applied to estimate the diagnostic performance of the MCP-1 and its accuracy to differentiate TD patients and healthy controls. *p* value <0.05 was regarded as statistically significant.

## Results

### Demographic characteristics

Age, gender, height, weight, waist circumference, hip circumference, and waist-to-hip ratios were significantly different among the four groups (*p* < 0.05). The TD group and T2DM group were older than the HC group, respectively (*p* < 0.05). There were more males in the T2DM group than in the MDD group, and more females in the MDD group than the T2DM group (*p* < 0.05). The T2DM group weighed more than the MDD and TD group, respectively (*p* < 0.05). The waist circumference of the T2DM group was larger than the MDD group (*p* < 0.05). Hip circumference in HC and T2DM groups was greater than MDD group, respectively (*p* < 0.05). The waist-to-hip ratio in the TD group was greater than that in the HC group (*p* < 0.05). There was no significant difference in the disease course, BMI, SBP, and DBP among the four groups, as shown in Table 1.

**Table 1:** Demographic characteristics of study participants (*n* = 84).

Variables	HC (*n* = 24)	T2DM (*n* = 21)	MDD (*n* = 23)	TD (*n* = 16)
Age (years)^c^	38.50 (34.00, 49.25) ^1*3**^	50.00 (43.00, 60.00)^1*^	45.50 (38.25, 55.25)	59.00 (41.00, 65.00)^3**^
Gender (male/female)^b^	12/12	16/5^4**^	8/15^4**^	10/6
Duration (years)^c^		3.00 (1.50, 7.50)	0.75 (0.33, 3.00)	4.50 (2.00, 8.00)
Height (m)^c^	1.63 (1.59, 1.68)	1.70 (1.65, 1.76)^4*^	1.61 (1.59, 1.70)^4*^	1.60 (1.70, 1.73)
Weight (kg)^a^	64.48 ± 10.94	70.60 ± 10.13^4*5*^	62.70 ± 10.81^4*^	62.25 ± 12.97^5*^
BMI (kg/㎡)^a^	23.84 ± 2.50	24.44 ± 2.50	23.34 + 3.22	23.21 ± 3.31
Waist circumference (cm)^a^	87.29 ± 10.20	94.31 ± 8.58^4*^	85.52 ± 14.25^4*^	91.38 ± 4.79
Hip circumference (cm)^c^	99 (93, 102)^2*^	100 (95.5, 104.25)^4***^	90 (84.75, 98)^2*4***^	95 (92, 99)
Waist-hip rate^c^	0.90 (0.83, 0.95)^3**^	0.93 (0.89, 0.98)	0.93 (0.85, 0.97)	0.95 (0.94, 0.98)^3**^
Systolic pressure (mmHg)^a^	120.88 ± 11.28	114.86 ± 21.37	114.52 ± 12.30	118.44 ± 12.79
Diastolic pressure (mmHg)^c^	84.50 (78.25, 91.75)	83.00 (70.00, 91.50)	81.00 (70.00, 85.00)	79.00 (69.00, 86.00)

^a^One-way ANOVA test ($\overset{?}{x}?s$), ^b^Chi-square test, ^c^Non-parametric Kruskal-Wallis test [M (P25, P75)]. ^1^Comparison between HC and T2DM, ^2^Comparison between HC and MDD, ^3^Comparison between HC and TD, ^4^Comparison between T2DM and MDD, ^5^Comparison between T2DM and TD, ^6^Comparison between MDD and TD; *p < 0.05; **p < 0.01; ***p < 0.001. HC, healthy control; T2DM, Type 2 diabetes; MDD, major depressive disorder; TD, T2DM combined with MDD; BMI, body mass index.

### Clinical characteristics

Fasting glucose, insulin resistance index, HbA1c, HDL, and LDL were significantly different among the four groups (*p* < 0.05). The fasting blood glucose levels were lower in the MDD group than in the HC, T2DM, and TD groups, respectively (*p* < 0.05). The insulin resistance index in the TD group was greater than that in the HC group, and greater insulin resistance index in T2DM than in HC and MDD groups, respectively (*p* < 0.05). There was a decreasing trend of insulin resistance index in the TD group compared to the T2DM group. The HbA1c levels were higher in the TD group than in the HC and MDD groups, and higher in the T2DM group than that in the HC and MDD groups, respectively (*p* < 0.05). There was a decreasing trend of HbA1c levels in the TD group compared to the T2DM group. The HDL levels were higher in the HC group than in the T2DM group (*p* < 0.05). The HC group had higher LDL levels than the MDD group (*p* < 0.05). There was no significant difference in fasting insulin and triglyceride among the four groups, as shown in Table 2.

**Table 2:** Comparison of clinical characteristics (*n* = 84).

Variables	HC (*n* = 24)	T2DM (*n* = 21)	MDD (*n* = 23)	TD (*n* = 16)
FBG (mmol/L)^b^	5.22 (5.04, 5.50)^2**^	7.80 (5.19, 9.95)^4***^	4.57 (4.06, 4.89)^2**4***6***^	6.37 (4.84, 8.17)^6***^
Fasting insulin (mU/L)^b^	6.62 (5.04, 11.24)	11.34 (7.66, 15.61)	9.53 (6.61, 11.50)	9.94 (5.68, 14.67)
IR^b^	1.61 (1.18, 2.63)^1**^	3.93 (2.20, 6.47)^1**4**^	1.86 (1.23, 2.51)^4**^	2.32 (1.56, 3.63)
HbA1c (%)^b^	5.50 (5.40, 5.78)^1***3***^	8.50 (7.00, 10.85)^1***4***^	5.60 (5.30, 5.95)^4***6***^	6.90 (6.10, 8.10)^3***6***^
TC (mmol/L)^a^	4.81 ± 0.86^2*^	4.59 ± 1.50	4.15 ± 0.60^2*^	4.73 ± 1.39
TG (mmol/L)^b^	1.67 (0.81, 2.43)	1.40 (1.04, 3.01)	1.51 (1.04, 2.08)	1.48 (1.18, 2.70)
HDL (mmol/L)^b^	1.20 (1.03, 1.44)^1**^	0.93 (0.82, 1.08)^1**^	1.11 (1.00, 1.23)	1.02 (0.71, 1.18)
LDL (mmol/L)^a^	3.08 ± 0.78^2**^	2.62 ± 1.14	2.42 ± 0.49^2**^	2.79 ± 1.17

^a^One-way ANOVA test ($\overset{?}{x}?s$), ^b^Non-parametric Kruskal-Wallis test [M (P25, P75)]. ^1^Comparison between HC and T2DM, ^2^Comparison between HC and MDD, ^3^Comparison between HC and TD, ^4^Comparison between T2DM and MDD, ^5^Comparison between T2DM and TD, ^6^Comparison between MDD and TD; *p < 0.05, **p < 0.01; ***p < 0.001. HC, healthy control; T2DM, Type 2 diabetes; MDD, major depressive disorder; TD, T2DM combined with MDD; FBG, fasting blood glucose; IR, insulin resistance index; HbA1c, glycosylated hemoglobin; TC, total cholesterol; TG, triglycerides; HDL, high-density lipoprotein; LDL, low-density lipoprotein.

### HAMD and HAMA assessment

There was a statistical difference in HAMD and HAMA scores among the four groups (*p* < 0.05). The HAMD and HAMA scores were higher in the TD group than in the HC and T2DM groups, respectively (*p* < 0.05), and the HAMD and HAMA scores were higher in the MDD group than in the HC and T2DM groups, respectively (*p* < 0.05). There was a decreasing trend of HAMD and HAMA scores in the TD group compared to the MDD group, as shown in Table 3.

**Table 3:** HAMD-17 and HAMA assessment scores (*n* = 84).

Variables	HC (*n* = 24)	T2DM (*n* = 21)	MDD (*n* = 23)	TD (*n* = 16)
HAMD-17^a^	1.50 (0.00, 3.00)^2***3***^	2.00 (1.00, 3.00)^4***5***^	28.00 (24.00, 32.00)^2***4***^	25.00 (20.00, 30.00)^3***5***^
HAMA^a^	1.00 (0.00, 3.00)^2***3***^	1.00 (0.00, 2.00)^4***5***^	22.00 (16.00, 26.00)^2***4***^	21.00 (13.00, 24.00)^3***5***^

Non-parametric Kruskal-Wallis test [M (P25, P75)]. 1 Comparison between HC and T2DM, 2 Comparison between HC and MDD, 3 Comparison between HC and TD, 4 Comparison between T2DM and MDD, 5 Comparison between T2DM and TD, 6 Comparison between MDD and TD; ***p<0.001. HC, healthy control; T2DM, Type 2 diabetes; MDD, major depressive disorder; TD, T2DM combined with MDD; HAMD-17, 17-item Hamilton Depression Scale; HAMA, Hamilton Anxiety Scale.

### Comparison of cognitive function

**Figure 1:**
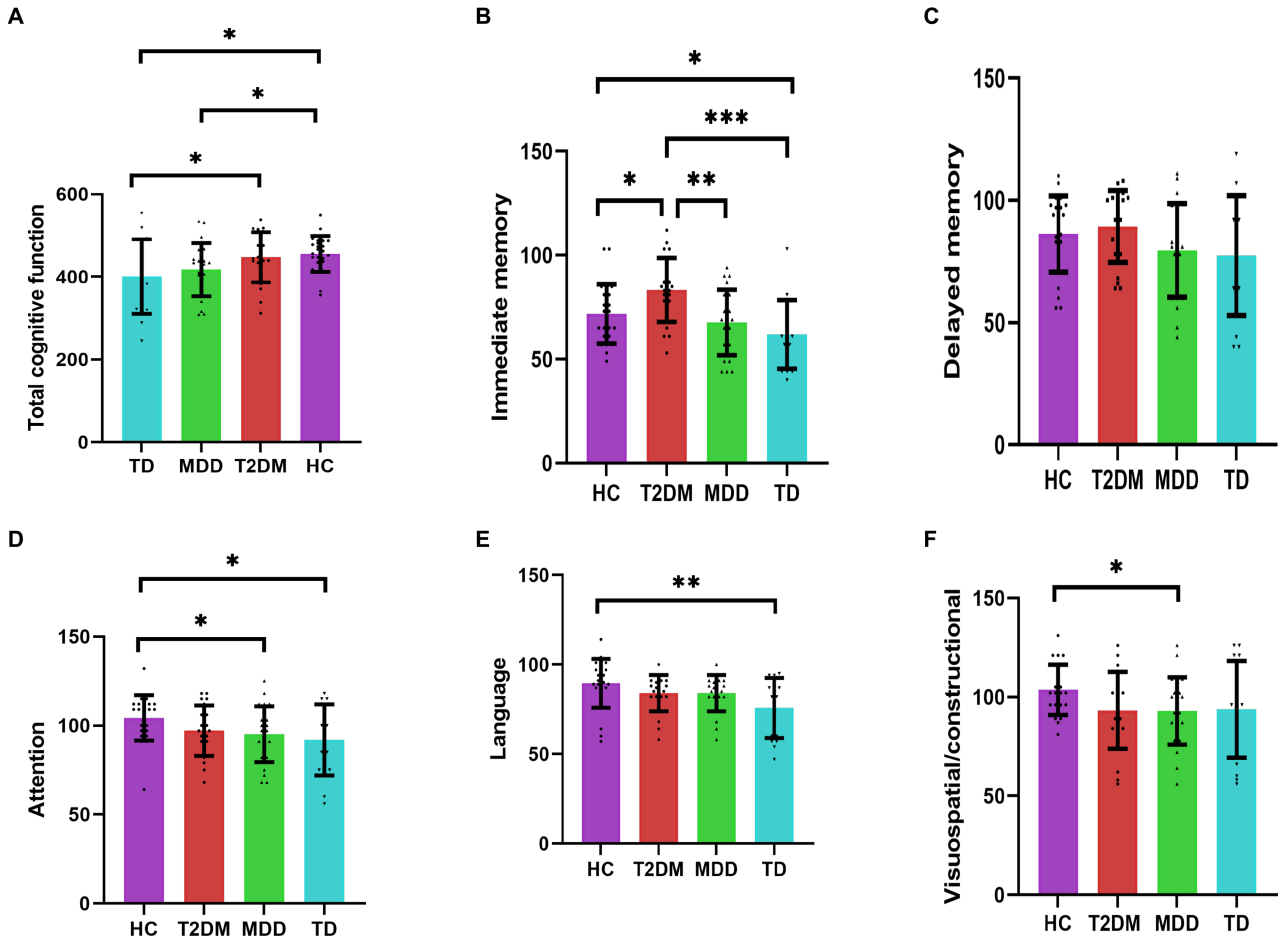
**(A–F)** Indicate the comparison of four groups for cognitive function (*n* = 84). *p < 0.05; **p < 0.01; ***p < 0.001. HC, healthy control; T2DM, Type 2 diabetes; MDD, major depressive disorder; TD, T2DM combined with MDD.

A statistically significant difference in total cognitive function scores, immediate memory, attention, language, and visuospatial/construction scores was observed among the four groups (*p* < 0.05). The total cognitive function scores in the TD group were lower than that in the HC group and T2DM group, respectively (*p* < 0.05); the total cognitive function scores were significantly lower in the MDD group than in the HC group (*p* < 0.05); the total cognitive function scores were lower in MDD group than in the T2DM group, and lower in the T2DM group than in the HC group, and lower in the TD group than in the MDD group, but the difference was not statistically significant (*p* > 0.05). The immediate memory scores were lower in the TD group than in the HC and T2DM groups, respectively (*p* < 0.05). Both MDD and HC groups had lower immediate memory scores than those in the T2DM group, respectively (*p* < 0.05). Both TD and MDD groups showed lower attention scores than the HC group, respectively (*p* < 0.05), lower in the T2DM group than the HC group, and higher than MDD and TD groups, respectively, but the difference was not statistically significant (*p* > 0.05). The language scores were lower in the TD group than in the HC group (*p* < 0.05), and lower than T2DM and MDD groups, respectively, but no significant difference (*p* > 0.05). Visuospatial/constructional scores were lower in the MDD group than in the HC group (*p* < 0.05). Visuospatial/constructional scores in the TD group were lower than the HC group and higher than T2DM and MDD groups, respectively, but no significant difference (*p* > 0.05). Statistical differences in delayed memory scores were not observed among the four groups. See Table 4; Figure 1.

**Table 4:** Cognitive function (*n* = 84).

Variables	HC (*n* = 24)	T2DM (*n* = 21)	MDD (*n* = 23)	TD (*n* = 16)
Total cognitive function^a^	455.58 ± 43.19^2*3*^	447.14 ± 60.54^5*^	417.65 ± 64.17 ^2*^	400.81 ± 89.84^3*5*^
Immediate memory^a^	71.79 ± 14.25^1*3*^	83.33 ± 15.34^1*4**5***^	67.70 ± 15.80 ^4**^	61.94 ± 16.47^3*5***^
Delayed memory^a^	86.29 ± 15.56	89.33 ± 14.67	79.61 ± 19.11	77.44 ± 24.53
Attention^a^	104.33 ± 12.75^2*3*^	97.14 ± 14.22	95.09 ± 15.70^2*^	91.94 ± 19.95^3*^
Language^a^	89.54 ± 13.61^3**^	84.10 ± 10.14	82.35 ± 17.18	75.75 ± 16.78^3**^
Visuospatial/constructional^a^	103.63 ± 12.63^2*^	93.24 ± 19.42	92.91 ± 16.96^2*^	93.75 ± 18.47

^a^One-way ANOVA test ($\overset{?}{x}?s$). ^1^Comparison between HC and T2DM, ^2^Comparison between HC and MDD, ^3^Comparison between HC and TD, ^4^Comparison between T2DM and MDD, ^5^Comparison between T2DM and TD, ^6^Comparison between MDD and TD; *p < 0.05, **p < 0.01; ***p < 0.001. HC, healthy control; T2DM, Type 2 diabetes; MDD, major depressive disorder; TD, T2DM combined with MDD.

### Expression of serum MCP-1

**Figure 2:**
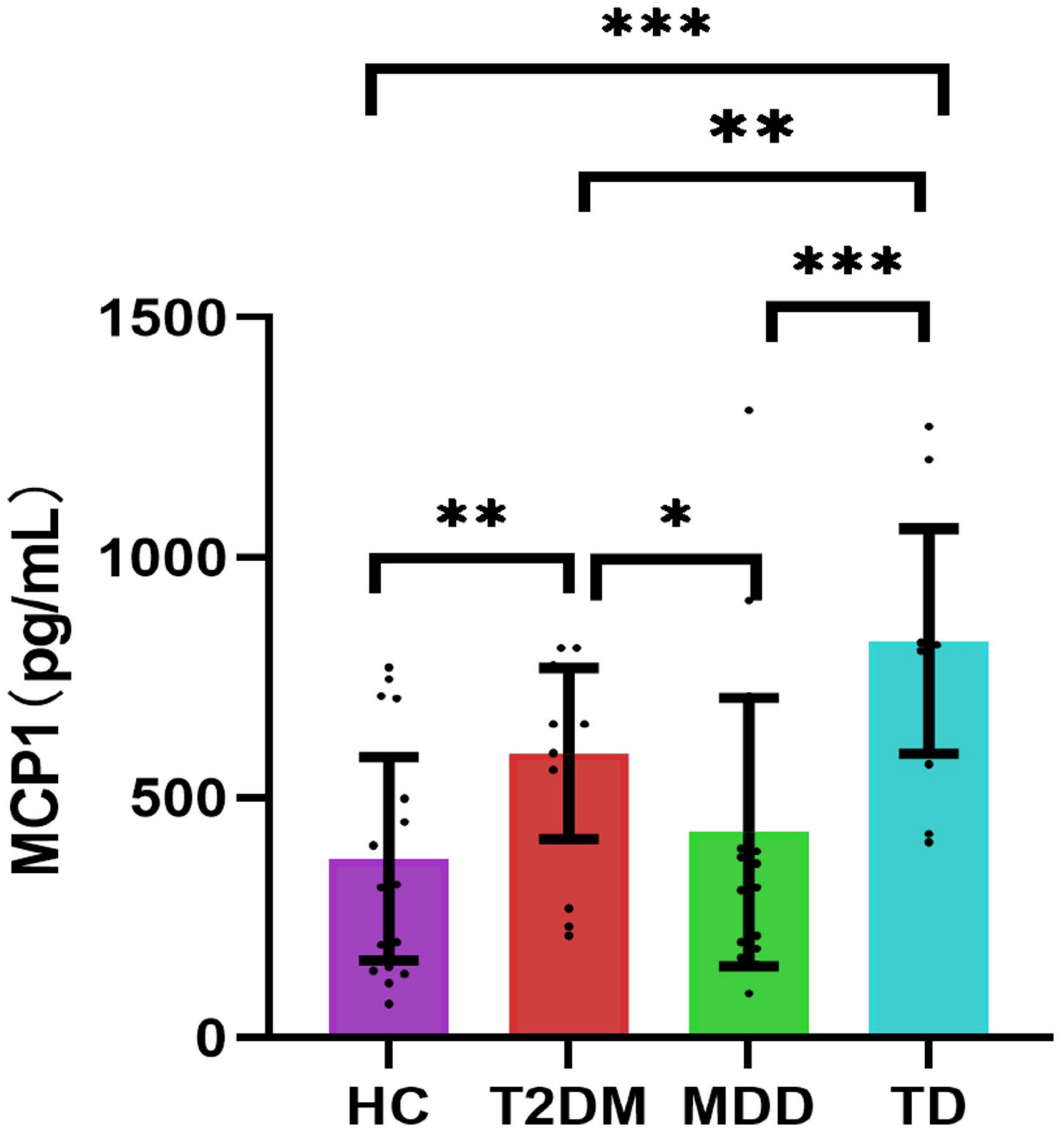
Comparisons of four groups for MCP1 [*n* = 84, M (P25, P75)]. *p < 0.05; **p < 0.01; ***p < 0.001. HC, healthy control; T2DM, Type 2 diabetes; MDD, major depressive disorder; TD, T2DM combined with MDD; MCP1, monocyte chemotactic protein-1.

The MCP-1 expression levels were statistically different among the four groups (*p* < 0.05). Serum MCP-1 levels were higher in the TD group compared with that in the HC, T2DM, and MDD groups, respectively (*p* < 0.05). Serum MCP-1 levels were higher in the T2DM group than that in the HC and MDD groups, respectively (*p* < 0.05), the MCP-1 levels were higher in the MDD group than that in the HC group, but there was no statistically significant difference, as shown in Table 5; Figure 2.

**Table 5:** Expression of MCP-1 (*n* = 84).

Variables	HC (*n* = 24)	T2DM (*n* = 21)	MDD (*n* = 23)	TD (*n* = 16)
MCP-1 ^a^	366.45 (172.94, 494.73)^1**3***^	611.57 (518.84, 741.54)^1**4*5**^	375.79 (212.07, 539.88)^4* 6***^	820.61 (728.35, 951.34)^3***5**6***^

^a^Non-parametric Kruskal-Wallis test [M (P25, P75)]. ^1^Comparison between HC and T2DM, ^2^Comparison between HC and MDD, ^3^Comparison between HC and TD, ^4^Comparison between T2DM and MDD, ^5^Comparison between T2DM and TD, ^6^Comparison between MDD and TD; *p < 0.05; **p < 0.01; ***p < 0.001. HC, healthy control; T2DM, Type 2 diabetes; MDD, major depressive disorder; TD, T2DM combined with MDD; MCP-1, monocyte chemotactic protein-1.

### Evaluation of diagnostic value for serum MCP-1 levels

**Figure 3:**
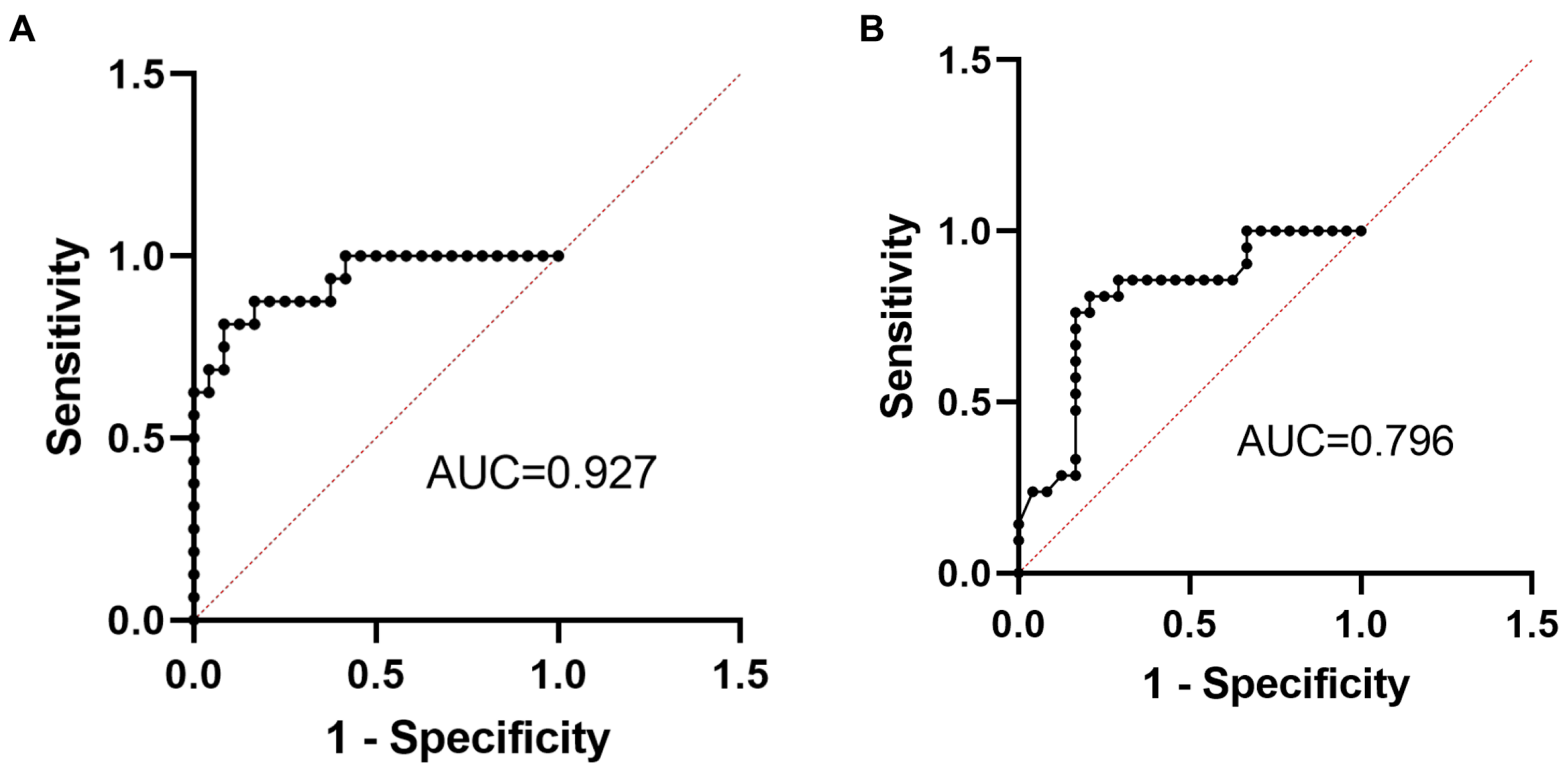
**(A,B)** Indicate the ROC curve for MCP-1 of TD group (*n* = 16) and T2DM group (*n* = 21), respectively. T2DM, Type 2 diabetes; TD, T2DM combined with MDD; ROC, the receiver operating characteristic curve; AUC, area under the curve; MCP-1, monocyte chemotactic protein-1.

To evaluate the performance of serum MCP-1 as predictive biomarkers for TD and T2DM groups, compared with the HC group, ROC curves were constructed. The discriminatory capability of the TD group had the highest area under the curve (AUC) of 0.9271 (cut-off point of 718.1 pg./mL, a sensitivity of 81.25% and specificity of 91.67%, *p* < 0.001), which was followed by T2DM group, with an AUC of 0.7956 (cut-off point of 503.8 pg./mL, sensitivity of 80.95% and a specificity of 79.17%, *p* = 0.007), as shown in Figure 3.

### Correlations between serum MCP-1 and clinical variables

The correlations between serum MCP-1 and clinical variables were performed. Serum MCP-1 levels were negatively correlated with a hip circumference in the T2DM group (*r* = −0.483; *p* = 0.027), but the correlation disappeared after adjusting age and gender (*r* = −0.372; *p* = 0.117). There were no such correlations between serum MPC-1 levels and other clinical variables (Table 6).

**Table 6:** Correlations between clinical variables and serum MCP-1 expression levels.

Variables	MCP-1 (TD, *n* = 16)	MCP-1 (T2DM, *n* = 21)	MCP-1 (MDD, *n* = 23)
Age	0.109 (*p* = 0.688)	−0.228 (*p* = 0.320)	0.039 (*p* = 0.860)
Gender	0.140 (*p* = 0.605)	0.250 (*p* = 0.275)	−0.303 (*p* = 0.160)
Duration	−0.208 (*p* = 0.439)	0.214 (*p* = 0.351)	−0.023 (*p* = 0.918)
Height	−0.333 (*p* = 0.207)	−0.092 (*p* = 0.691)	0.147 (*p* = 0.503)
Body weight	−0.395 (*p* = 0.130)	−0.267 (*p* = 0.241)	0.374 (*p* = 0.079)
BMI	−0.189 (*p* = 0.483)	−0.338 (*p* = 0.135)	0.341 (*p* = 0.112)
Waist circumference	0.056 (*p* = 0.837)	−0.362 (*p* = 0.107)	0.082 (*p* = 0.710)
Hip circumference	−0.035 (*p* = 0.896)	−0.483 (*p* = 0.027)^*^	0.212 (*p* = 0.332)
Waist-to-hip ratio	0.171 (*p* = 0.0.527)	0.020 (*p* = 0.931)	0.053 (*p* = 0.808)
Systolic pressure	0.012 (*p* = 0.965)	−0.081 (*p* = 0.726)	−0.165 (*p* = 0.451)
Diastolic pressure	−0.202 (*p* = 0.453)	−0.072 (*p* = 0.755)	−0.135 (*p* = 0.538)
FBG	−0.274 (*p* = 0.305)	0.135 (*p* = 0.561)	−0.053 (*p* = 0.811)
Fasting insulin	−0.121 (*p* = 0.656)	0.209 (*p* = 0.364)	−0.038 (*p* = 0.865)
IR	−0.218 (*p* = 0.418)	0.313 (*p* = 0.168)	0.043 (*p* = 0.847)
HbA1c	0.006 (*p* = 0.983)	0.076 (*p* = 0.743)	−0.229 (*p* = 0.293)
TC	−0.040 (*p* = 0.882)	0.090 (*p* = 0.700)	−0.069 (*p* = 0.755)
TG	−0.016 (*p* = 0.953)	−0.134 (*p* = 0.561)	−0.385 (*p* = 0.070)
HDL	−0.091 (*p* = 0.736)	0.264 (*p* = 0.248)	−0.118 (*p* = 0.593)
LDL	−0.093 (*p* = 0.731)	0.055 (*p* = 0.814)	0.060 (*p* = 0.785)

Spearman correlation analysis was used. *p < 0.05. HC, healthy control; T2DM, Type 2 diabetes; MDD, major depressive disorder; TD, T2DM combined with MDD; MCP-1, monocyte chemotactic protein-1; BMI, body mass index; FBG, fasting blood glucose; IR, insulin resistance index; HbA1c, glycosylated hemoglobin; TC, total cholesterol; TG, triglycerides; HDL, high-density lipoprotein; LDL, low-density lipoprotein.

### Correlations between serum MCP-1 and depression/anxiety/cognitive function

Correlation analysis was performed to find correlations between serum MCP-1 and depression/anxiety/cognitive function variables. But significant correlations were not observed between serum MPC-1 levels and depression/anxiety/cognitive function variables, as shown in Table 7.

**Table 7:** Correlations between depression/anxiety/cognitive function variables and serum MCP-1.

Variables	TD (*n* = 16)	T2DM (*n* = 21)	MDD (*n* = 23)
HAMD-17	−0.003 (*p* = 0.991)	−0.073 (*p* = 0.752)	0.195 (*p* = 0.373)
HAMA	0.310 (*p* = 0.243)	−0.014 (*p* = 0.950)	0.104 (*p* = 0.638)
Total cognitive function	0.194 (*p* = 0.471)	0.214 (*p* = 0.353)	0.134 (*p* = 0.542)
Immediate memory	0.215 (*p* = 0.424)	0.094 (*p* = 0.685)	0.224 (*P* = 0.305)
Delayed memory	0.157 (*p* = 0.562)	0.163 (*p* = 0.480)	0.158 (*p* = 0.471)
Attention	0.293 (*p* = 0.271)	0.225 (*p* = 0.328)	0.091 (*p* = 0.680)
Language	0.191 (*p* = 0.479)	0.175 (*p* = 0.447)	0.067 (*p* = 0.763)
Visuospatial/constructional	0.041 (*p* = 0.879)	0.212 (*p* = 0.355)	0.031 (*p* = 0.889)

Spearman correlation analysis was used. HC, healthy control; T2DM, Type 2 diabetes; MDD, major depressive disorder; TD, T2DM combined with MDD; HAMD-17, 17-item Hamilton Depression Scale; HAMA, Hamilton Anxiety Scale.

## Discussion

### TD patients had more serious cognitive impairment

T2DM is often comorbid MDD, and the effects between them are usually bidirectional. These effects may be related to glycemic control, insulin resistance, blood pressure control, etc. (Moran et al., 2022). A previous follow-up study found that depression scores of T2DM were higher than those of healthy controls, patients with diabetes had a higher prevalence of depression with longer duration, and depressive symptoms of diabetes patients with shorter duration were not significantly different from those of healthy controls (Rensma et al., 2020). Another study showed that higher depressive symptom scores reflected heavier metabolic syndrome severity in women and that both depressive symptom scores and metabolic syndrome severity scores were associated with lower physical activity levels and higher C-reactive protein levels (Gurka et al., 2016). In our study, the depression and anxiety scores were significantly higher in the TD and MDD groups, and the depression scores in the T2DM group showed an elevated trend compared with the HC group, which was consistent with the results of the previous study.

In our study, the total cognitive function scores were significantly lower in both MDD and TD groups, and the scores of the TD group were the lowest, indicating that both comorbidities aggravated the cognitive dysfunction, which is consistent with the conclusions of previous studies (Katon et al., 2015; Demakakos et al., 2017). Studies have found that patients with T2DM or MDD had a significantly lower executive function, while patients with comorbidity were more significantly severe than those with T2DM or MDD alone, and the aggravation of diabetes and depressive symptoms was negatively correlated with memory and executive function (Hamer et al., 2019), which suggested that the severe cognitive impairment was associated with increased blood glucose and aggravated depressive symptoms.

The cause of the aggravated cognitive impairment in the TD group may be related to the following two factors. On the one hand, depressive disorder is associated with the increased risk of cognitive decline, metabolic dysregulation, cardiovascular disease morbidity, mortality, the accelerated rate of cellular aging, and age-related diseases such as brain aging (Verhoeven et al., 2018; John et al., 2019; Han and Ham, 2021). A greater number of depressive symptoms were related to lower total cognitive function, executive function, semantic classification, and episodic memory scores, while more emotional blunting was also associated with a faster decline in executive function (Soleimani et al., 2021). The increase in depressive symptoms over time was associated with parallel cognitive decline, suggesting that the natural course of depressive disorders and cognitive dysfunction progressed simultaneously, with possible common underlying mechanisms (Ravona-Springer et al., 2021). On the other hand, patients with T2DM were at increased risk of mild cognitive impairment and dementia (Anita et al., 2022). A survey of community-dwelling elderly residents showed that T2DM was associated with decreased nonverbal memory and fluency after 5 years. Patients with T2DM may already have lower attentional processing speed, visuospatial capacity, and visual memory at baseline, and there was a trend toward a greater decline in verbal fluency, verbal memory, and working memory during subsequent assessments. There were significant interactions between verbal memory/verbal fluency of diabetes patients and time after adjusting for age, gender, education, and vascular risk factors (Callisaya et al., 2019). In our study, the cognitive function scores of the T2DM group were lower than that of the HC group, but not statistically significant, which may be related to the short course, first-episode diabetes, and insufficient time interaction. In the future, the long-range changes of diabetes should be observed to clarify the aggravation of cognitive impairment in T2DM patients.

### Patients with TD have significantly higher MCP-1 expression levels, and MCP-1 may have a good diagnostic value for TD

It was indicated that MCP-1 may play a key role in insulin resistance, type 2 diabetes, depression, and Type 2 diabetes comorbid depression through oxidative stress and immune response, and so on. For example, MCP-1 act as a regulator in the polarization of Th0 cells toward the Th2 phenotype, which may result in the enhancement of immune response. Some studies showed that oxidative stress and immune response have been associated with type 2 diabetes, depression, and even TD, so MCP-1 may contribute to them by some mechanisms (Koth et al., 2004; Singh et al., 2021).

Inflammation is involved in the development and progression of MDD. And MDD is confirmed as a proinflammatory state. Increased proinflammatory factors can be potential biomarkers for MDD in the future (Osimo et al., 2020; Carniel and da Rocha, 2021). As neuroregulatory factors, proinflammatory factors are the key factors in the regulation of behavioral, neuroendocrine, and neurochemical characteristics of MDD. Treating cancer or hepatitis C with proinflammatory factors can induce depressive symptoms, while anti-inflammatory medication can relieve depressive symptoms (He et al., 2020). Studies have found that non-steroidal anti-inflammatory medication can reduce depressive symptoms, and increase response and remission rates in MDD patients (Bai et al., 2020). MCP-1 levels were significantly higher in the MDD group than in the HC group before medication treatment and significantly decreased after being given antidepressant treatment (de la Peña et al., 2020). And MCP-1 levels were significantly elevated in MDD patients with anxiety (Gaspersz et al., 2017). Increased levels of MCP-1 were also associated with reduced psychomotor speed in MDD patients (Goldsmith et al., 2016). In our study, MCP-1 expression levels tended to increase in the MDD group compared with the HC group, but there was no statistical difference, possibly due to the small sample size. Our study also found that the TD group had the highest expression levels of MCP-1, suggesting that comorbidities may aggravate the inflammatory process. In addition, the total RBANS scores, immediate memory, attention, and language scores also decreased significantly in the TD group, and there was also a downward trend in the immediate memory and visuospatial/constructional scores, suggesting that inflammation may play an important role in the cognitive impairment of TD, which is also consistent with the results of previous studies about inflammation. Previous studies found that T2DM patients with strong loneliness had increased MCP-1 levels after acute stress (Hackett et al., 2019). Other proinflammatory factors, including high-sensitivity CRP and IL-1, increased significantly in patients with TD, compared with T2DM patients without depression, and depression scores were positively correlated with vascular endothelial growth factor, IL-1b, and MCP-1, respectively (Laake et al., 2014). Higher levels of serum MCP-1 at baseline were associated with longitudinal declines in overall cognitive scores and episodic memory performance in older adults during 4-year follow-ups (Ehses et al., 2009). Higher levels of MCP-1 and IL-6 were associated with poorer executive cognitive function respectively, and higher levels of MCP-1 were associated with worse episodic memory (Stenfors et al., 2017). In a meta-analysis study, Su et al. (2019) found that Alzheimer’s patients had increased MCP-1 and MCP3 levels, and MCP-1 levels were also elevated in patients with mild cognitive dysfunction. There was no significant association between MCP-1 and cognitive function in the TD group in our study, which may be related to the small study sample size. In addition, our study also showed that MCP-1 had an optimal TD diagnosis value with a sensitivity and specificity of 0.82 and 0.92 (AUC = 0.93), respectively, which may be a useful biomarker for the diagnosis of TD in the future.

Increased infiltration of macrophages and other immune cells in the adipose tissue, liver, muscle, and pancreas in T2DM patients can stimulate the production of proinflammatory factors, interfere with the insulin signaling in peripheral tissues, or induce β cellular dysfunction and subsequent insulin deficiency, which leads to the development of T2DM (Ehses et al., 2009; Donath and Shoelson, 2011; Esser et al., 2014). Our study showed that MCP-1 levels were higher in the T2DM group than in the HC group, which is consistent with previous studies. Some studies found that higher MCP-1 levels were associated with insulin resistance in the T2DM group (Degirmenci et al., 2019), but no such association in our study. In addition, MCP-1 was also associated with the complications of T2DM. For example, MCP-1 and vascular endothelial growth factor significantly increased in patients with diabetic retinopathy bleeding compared with T2DM patients without retinopathy bleeding (Ra et al., 2021). In addition, compared with the patients without diabetic neuropathy, the T2DM patients with diabetic neuropathy had significantly higher MCP-1 and IL-8 levels (Mussa et al., 2021). MCP-1 expression was also significantly associated with the risk of early decline in renal function (Nowak et al., 2018). The urinary microalbumin of T2DM patients consistently increased within a 6.5-year follow-up, with increasing serum and urinary MCP-1 levels, suggesting that MCP-1 may be a biomarker of early diabetic nephropathy (Ehses et al., 2009). Furthermore, elevated systemic inflammation led to an increased risk of diabetes-associated cognitive impairment or dementia, thus inflammation may be associated with a greater risk of the development and progression of cognitive dysfunction in patients with T2DM (Anita et al., 2022). What’s more, the ROC curve of our study also showed that the optimal sensitivity and specificity of MCP-1 for diagnosing T2DM were 0.82 and 0.79, respectively, with AUC = 0.80. So MCP-1 may be one of the effective biomarkers of T2DM in the future.

There are some limitations in our study. First, the age, gender, and course of medication of patients are not controlled, which may affect the research results. Second, the participants have a large age span, which may be an influencing factor. Thirdly, small sample size and only a cross-sectional study may affect the study findings. Therefore, we need a large sample, a long-term follow-up study in the future, and strict control of age, gender, and medication. Also, animal experiments should be performed to verify the specific pathophysiological mechanism of TD in the future.

## Data availability statement

The original contributions presented in the study are included in the article/supplementary material, further inquiries can be directed to the corresponding author.

## Ethics statement

The studies involving human participants were reviewed and approved by the Ethics Committee of the First Affiliated hospital of Kunming Medical University. The patients/participants provided their written informed consent to participate in this study.

## Author contributions

FC contributed to the conception, organization, execution, data collection and statistical analysis, and drafting of the manuscript. XZ and YC contributed to the execution and data collection. MY and LS contributed to the conception, organization, execution, and data collection. NL contributed to the conception and organization, manuscript review and critique, and was responsible for the overall content as the guarantor. All authors contributed to the article and approved the submitted version.

## Funding

This study was supported by the study of relationship between the expression of vascular growth factor and the prognosis of type 2 diabetes mellitus combined with major depressive disorder (Grant No. 202001AY070001-035); The National Natural Science Foundation of China (Grant No. 82160164);Yunnan Clinical Research Center for Mental Diseases(Grant No. 0105679005).

## Conflict of interest

The authors declare that the research was conducted in the absence of any commercial or financial relationships that could be construed as a potential conflict of interest.

## Publisher’s note

All claims expressed in this article are solely those of the authors and do not necessarily represent those of their affiliated organizations, or those of the publisher, the editors and the reviewers. Any product that may be evaluated in this article, or claim that may be made by its manufacturer, is not guaranteed or endorsed by the publisher.

## References

[ref1] American Diabetes Association (2020). 2. Classification and diagnosis of diabetes: standards of medical Care in Diabetes-2020. Diabetes Care 43, S14–S31. doi: 10.2337/dc20-S002, PMID: 31862745

[ref2] Anita N. Z. Zebarth J. Chan B. Wu C. Y. Syed T. Shahrul D.. (2022). Inflammatory markers in type 2 diabetes with vs. without cognitive impairment; a systematic review and meta-analysis. Brain Behav. Immun. 100, 55–69. doi: 10.1016/j.bbi.2021.11.005, PMID: 34808290

[ref3] Bai S. Guo W. Feng Y. Deng H. Li G. Nie H.. (2020). Efficacy and safety of anti-inflammatory agents for the treatment of major depressive disorder: a systematic review and meta-analysis of randomised controlled trials. J. Neurol. Neurosurg. Psychiatry 91, 21–32. doi: 10.1136/jnnp-2019-320912, PMID: 31658959

[ref4] Biessels G. J. Whitmer R. A. (2020). Cognitive dysfunction in diabetes: how to implement emerging guidelines. Diabetologia 63, 3–9. doi: 10.1007/s00125-019-04977-9, PMID: 31420699 PMC6890615

[ref5] Callisaya M. L. Beare R. Moran C. Phan T. Wang W. Srikanth V. K. (2019). Type 2 diabetes mellitus, brain atrophy and cognitive decline in older people: a longitudinal study. Diabetologia 62, 448–458. doi: 10.1007/s00125-018-4778-9, PMID: 30547230

[ref6] Carniel B. P. da Rocha N. S. (2021). Brain-derived neurotrophic factor (BDNF) and inflammatory markers: perspectives for the management of depression. Prog. Neuro-Psychopharmacol. Biol. Psychiatry 108:110151. doi: 10.1016/j.pnpbp.2020.110151, PMID: 33096156

[ref7] Castro-Costa E. Diniz B. S. Firmo J. Peixoto S. V. de Loyola Filho A. I. Lima-Costa M. F.. (2019). Diabetes, depressive symptoms, and mortality risk in old age: the role of inflammation. Depress. Anxiety 36, 941–949. doi: 10.1002/da.22908, PMID: 31066979

[ref8] de la Peña F. R. Cruz-Fuentes C. Palacios L. Girón-Pérez M. I. Medina-Rivero E. Ponce-Regalado M. D.. (2020). Serum levels of chemokines in adolescents with major depression treated with fluoxetine. World J. Psychiatry. 10, 175–186. doi: 10.5498/wjp.v10.i8.175, PMID: 32874955 PMC7439300

[ref9] Degirmenci I. Ozbayer C. Kebapci M. N. Kurt H. Colak E. Gunes H. V. (2019). Common variants of genes encoding TLR4 and TLR4 pathway members TIRAP and IRAK1 are effective on MCP-1, IL6, IL1β, and TNFα levels in type 2 diabetes and insulin resistance. Inflamm. Res. 68, 801–814. doi: 10.1007/s00011-019-01263-7, PMID: 31222667

[ref10] Demakakos P. Muniz-Terrera G. Nouwen A. (2017). Type 2 diabetes, depressive symptoms and trajectories of cognitive decline in a national sample of community-dwellers: a prospective cohort study. PLoS One 12:e0175827. doi: 10.1371/journal.pone.0175827, PMID: 28414754 PMC5393617

[ref11] Donath M. Y. (2014). Targeting inflammation in the treatment of type 2 diabetes: time to start. Nat. Rev. Drug Discov. 13, 465–476. doi: 10.1038/nrd4275, PMID: 24854413

[ref12] Donath M. Y. Shoelson S. E. (2011). Type 2 diabetes as an inflammatory disease. Nat. Rev. Immunol. 11, 98–107. doi: 10.1038/nri2925 21233852

[ref13] Ehses J. A. Ellingsgaard H. Böni-Schnetzler M. Donath M. Y. (2009). Pancreatic islet inflammation in type 2 diabetes: from alpha and beta cell compensation to dysfunction. Arch. Physiol. Biochem. 115, 240–247. doi: 10.1080/13813450903025879, PMID: 19645635

[ref14] Esser N. Legrand-Poels S. Piette J. Scheen A. J. Paquot N. (2014). Inflammation as a link between obesity, metabolic syndrome and type 2 diabetes. Diabetes Res. Clin. Pract. 105, 141–150. doi: 10.1016/j.diabres.2014.04.006 24798950

[ref15] Feil D. G. Rajan M. Soroka O. Tseng C. L. Miller D. R. Pogach L. M. (2011). Risk of hypoglycemia in older veterans with dementia and cognitive impairment: implications for practice and policy. J. Am. Geriatr. Soc. 59, 2263–2272. doi: 10.1111/j.1532-5415.2011.03726.x, PMID: 22150156

[ref16] Ferland-McCollough D. Maselli D. Spinetti G. Sambataro M. Sullivan N. Blom A.. (2018). MCP-1 feedback loop between adipocytes and mesenchymal stromal cells causes fat accumulation and contributes to hematopoietic stem cell rarefaction in the bone marrow of patients with diabetes. Diabetes 67, 1380–1394. doi: 10.2337/db18-0044, PMID: 29703845

[ref17] Gaspersz R. Lamers F. Wittenberg G. Beekman A. van Hemert A. M. Schoevers R. A.. (2017). The role of anxious distress in immune dysregulation in patients with major depressive disorder. Transl. Psychiatry 7:1268. doi: 10.1038/s41398-017-0016-3, PMID: 29217840 PMC5802575

[ref18] Goldsmith D. R. Haroon E. Woolwine B. J. Jung M. Y. Wommack E. C. Harvey P. D.. (2016). Inflammatory markers are associated with decreased psychomotor speed in patients with major depressive disorder. Brain Behav. Immun. 56, 281–288. doi: 10.1016/j.bbi.2016.03.025, PMID: 27040122 PMC4939278

[ref19] Gurka M. J. Vishnu A. Okereke O. I. Musani S. Sims M. DeBoer M. D. (2016). Depressive symptoms are associated with worsened severity of the metabolic syndrome in African American women independent of lifestyle factors: a consideration of mechanistic links from the Jackson heart study. Psychoneuroendocrinology 68, 82–90. doi: 10.1016/j.psyneuen.2016.02.030, PMID: 26963374 PMC5105331

[ref20] Hackett R. A. Poole L. Hunt E. Panagi L. Steptoe A. (2019). Loneliness and biological responses to acute stress in people with type 2 diabetes. Psychophysiology 56:e13341. doi: 10.1111/psyp.13341, PMID: 30693534 PMC6563153

[ref21] Hamer J. A. Testani D. Mansur R. B. Lee Y. Subramaniapillai M. McIntyre R. S. (2019). Brain insulin resistance: a treatment target for cognitive impairment and anhedonia in depression. Exp. Neurol. 315, 1–8. doi: 10.1016/j.expneurol.2019.01.016, PMID: 30695707

[ref22] Han K. M. Ham B. J. (2021). How inflammation affects the brain in depression: a review of functional and structural MRI studies. J. Clin. Neurol. 17, 503–515. doi: 10.3988/jcn.2021.17.4.503, PMID: 34595858 PMC8490908

[ref23] He Y. Li W. Wang Y. Tian Y. Chen X. Wu Z.. (2020). Major depression accompanied with inflammation and multiple cytokines alterations: evidences from clinical patients to macaca fascicularis and LPS-induced depressive mice model. J. Affect. Disord. 271, 262–271. doi: 10.1016/j.jad.2020.03.131, PMID: 32479325

[ref24] Hotamisligil G. S. (2017). Inflammation, metaflammation and immunometabolic disorders. Nature 542, 177–185. doi: 10.1038/nature21363, PMID: 28179656

[ref25] John A. Patel U. Rusted J. Richards M. Gaysina D. (2019). Affective problems and decline in cognitive state in older adults: a systematic review and meta-analysis. Psychol. Med. 49, 353–365. doi: 10.1017/S0033291718001137, PMID: 29792244 PMC6331688

[ref26] Katon W. Pedersen H. S. Ribe A. R. Fenger-Grøn M. Davydow D. Waldorff F. B.. (2015). Effect of depression and diabetes mellitus on the risk for dementia: a national population-based cohort study. JAMA Psychiat. 72, 612–619. doi: 10.1001/jamapsychiatry.2015.0082 PMC4666533 25875310

[ref27] Khaledi M. Haghighatdoost F. Feizi A. Aminorroaya A. (2019). The prevalence of comorbid depression in patients with type 2 diabetes: an updated systematic review and meta-analysis on huge number of observational studies. Acta Diabetol. 56, 631–650. doi: 10.1007/s00592-019-01295-9, PMID: 30903433

[ref28] Kher M. Beri S. Rehan H. S. Prakash A. Gupta L. K. (2020). Effect of metformin and insulin combination on monocyte chemoattractant protein-1 and cathepsin-D in type 2 diabetes mellitus. Diabetes Metab. Syndr. 14, 1703–1710. doi: 10.1016/j.dsx.2020.08.016, PMID: 32911202

[ref29] Koth L. L. Rodriguez M. W. Bernstein X. L. Chan S. Huang X. Charo I. F.. (2004). Aspergillus antigen induces robust Th2 cytokine production, inflammation, airway hyperreactivity and fibrosis in the absence of MCP-1 or CCR2. Respir. Res. 5:12. doi: 10.1186/1465-9921-5-12, PMID: 15377395 PMC520828

[ref30] Laake J. P. Stahl D. Amiel S. A. Petrak F. Sherwood R. A. Pickup J. C.. (2014). The association between depressive symptoms and systemic inflammation in people with type 2 diabetes: findings from the South London diabetes study. Diabetes Care 37, 2186–2192. doi: 10.2337/dc13-2522, PMID: 24842983

[ref31] Lee W. J. Liao Y. C. Wang Y. F. Lin I. F. Wang S. J. Fuh J. L. (2018). Plasma MCP-1 and cognitive decline in patients with Alzheimer's disease and mild cognitive impairment: a two-year follow-up study. Sci. Rep. 8:1280. doi: 10.1038/s41598-018-19807-y, PMID: 29352259 PMC5775300

[ref32] Moran C. Than S. Callisaya M. Beare R. Srikanth V. (2022). New horizons-cognitive dysfunction associated with type 2 diabetes. J. Clin. Endocrinol. Metab. 107, 929–942. doi: 10.1210/clinem/dgab797, PMID: 34788847

[ref33] Mussa B. M. Srivastava A. Al-Habshi A. Mohammed A. K. Halwani R. Abusnana S. (2021). Inflammatory biomarkers levels in T2DM Emirati patients with diabetic neuropathy. Diabetes Metab. Syndr. Obes. 14, 3389–3397. doi: 10.2147/DMSO.S319863, PMID: 34345175 PMC8323777

[ref34] Muzurović E. Stanković Z. Kovačević Z. Škrijelj B. Š. Mikhailidis D. P. (2021). Inflammatory markers associated with diabetes mellitus - old and new players. Curr. Pharm. Des. 27, 3020–3035. doi: 10.2174/1381612826666201125103047, PMID: 33238871

[ref35] Nguyen M. M. Perlman G. Kim N. Wu C. Y. Daher V. Zhou A.. (2021). Depression in type 2 diabetes: a systematic review and meta-analysis of blood inflammatory markers. Psychoneuroendocrinology 134:105448. doi: 10.1016/j.psyneuen.2021.105448, PMID: 34687965

[ref36] Nowak N. Skupien J. Smiles A. M. Yamanouchi M. Niewczas M. A. Galecki A. T.. (2018). Markers of early progressive renal decline in type 2 diabetes suggest different implications for etiological studies and prognostic tests development. Kidney Int. 93, 1198–1206. doi: 10.1016/j.kint.2017.11.024, PMID: 29398132 PMC5911430

[ref37] Olaithe M. Weinborn M. Lowndes T. Ng A. Hodgson E. Fine L.. (2019). Repeatable battery for the assessment of neuropsychological status (RBANS): normative data for older adults. Arch. Clin. Neuropsychol. 34, 1356–1366. doi: 10.1093/arclin/acy102, PMID: 30608541

[ref38] Osimo E. F. Pillinger T. Rodriguez I. M. Khandaker G. M. Pariante C. M. Howes O. D. (2020). Inflammatory markers in depression: a meta-analysis of mean differences and variability in 5,166 patients and 5,083 controls. Brain Behav. Immun. 87, 901–909. doi: 10.1016/j.bbi.2020.02.010, PMID: 32113908 PMC7327519

[ref39] Ra H. Lee A. Lee J. Kim I. Baek J. (2021). Cytokines associated with hemorrhage in proliferative diabetic retinopathy. Int. Ophthalmol. 41, 1845–1853. doi: 10.1007/s10792-021-01746-9, PMID: 33609201

[ref40] Rauwerda N. L. Tovote K. A. Peeters A. Sanderman R. Emmelkamp P. Schroevers M. J.. (2018). WHO-5 and BDI-II are acceptable screening instruments for depression in people with diabetes. Diabet. Med. 35, 1678–1685. doi: 10.1111/dme.13779, PMID: 30019352

[ref41] Ravona-Springer R. Heymann A. Lin H. M. Liu X. Berman Y. Schwartz J.. (2021). Increase in number of depression symptoms over time is related to worse cognitive outcomes in older adults with type 2 diabetes. Am. J. Geriatr. Psychiatry 29, 1–11. doi: 10.1016/j.jagp.2020.09.022, PMID: 33127316 PMC7771631

[ref42] Reddy S. Amutha A. Rajalakshmi R. Bhaskaran R. Monickaraj F. Rangasamy S.. (2017). Association of increased levels of MCP-1 and cathepsin-D in young onset type 2 diabetes patients (T2DM-Y) with severity of diabetic retinopathy. J. Diabetes Complicat. 31, 804–809. doi: 10.1016/j.jdiacomp.2017.02.017, PMID: 28336215

[ref43] Rensma S. P. van Sloten T. T. Ding J. Sigurdsson S. Stehouwer C. Gudnason V.. (2020). Type 2 diabetes, change in depressive symptoms over time, and cerebral small vessel disease: longitudinal data of the AGES-Reykjavik study. Diabetes Care 43, 1781–1787. doi: 10.2337/dc19-2437, PMID: 32527799 PMC7372064

[ref44] Schrauben S. J. Shou H. Zhang X. Anderson A. H. Bonventre J. V. Chen J.. (2021). Association of Multiple Plasma Biomarker Concentrations with progression of prevalent diabetic kidney disease: findings from the chronic renal insufficiency cohort (CRIC) study. J. Am. Soc. Nephrol. 32, 115–126. doi: 10.1681/ASN.2020040487, PMID: 33122288 PMC7894671

[ref45] Singh S. Anshita D. Ravichandiran V. (2021). MCP-1: function, regulation, and involvement in disease. Int. Immunopharmacol. 101:107598. doi: 10.1016/j.intimp.2021.107598, PMID: 34233864 PMC8135227

[ref46] Sluiman A. J. McLachlan S. Forster R. B. Strachan M. Deary I. J. Price J. F. (2022). Higher baseline inflammatory marker levels predict greater cognitive decline in older people with type 2 diabetes: year 10 follow-up of the Edinburgh type 2 diabetes study. Diabetologia 65, 467–476. doi: 10.1007/s00125-021-05634-w, PMID: 34932135 PMC8803673

[ref47] Soleimani L. Ravona-Springer R. Lin H. M. Liu X. Sano M. Heymann A.. (2021). Specific dimensions of depression have different associations with cognitive decline in older adults with type 2 diabetes. Diabetes Care 44, 655–662. doi: 10.2337/dc20-2031, PMID: 33468519 PMC7896256

[ref48] Stenfors C. Jonsdottir I. H. Magnusson Hanson L. L. Theorell T. (2017). Associations between systemic pro-inflammatory markers, cognitive function and cognitive complaints in a population-based sample of working adults. J. Psychosom. Res. 96, 49–59. doi: 10.1016/j.jpsychores.2017.03.010, PMID: 28545793

[ref49] Su C. Zhao K. Xia H. Xu Y. (2019). Peripheral inflammatory biomarkers in Alzheimer's disease and mild cognitive impairment: a systematic review and meta-analysis. Psychogeriatrics 19, 300–309. doi: 10.1111/psyg.12403, PMID: 30790387

[ref50] Verhoeven J. E. Révész D. Picard M. Epel E. E. Wolkowitz O. M. Matthews K. A.. (2018). Depression, telomeres and mitochondrial DNA: between- and within-person associations from a 10-year longitudinal study. Mol. Psychiatry 23, 850–857. doi: 10.1038/mp.2017.48, PMID: 28348385

[ref51] Wang K. Li F. Cui Y. Cui C. Cao Z. Xu K.. (2019). The association between depression and type 1 diabetes mellitus: inflammatory cytokines as ferrymen in between? Mediat. Inflamm. 2019:2987901. doi: 10.1155/2019/2987901, PMID: 31049023 PMC6458932

[ref52] Wang W. Y. Zheng Y. S. Li Z. G. Cui Y. M. Jiang J. C. (2019). MiR-92a contributes to the cardiovascular disease development in diabetes mellitus through NF-κB and downstream inflammatory pathways. Eur. Rev. Med. Pharmacol. Sci. 23, 3070–3079. doi: 10.26355/eurrev_201904_17589, PMID: 31002156

